# Characterization of a Novel M4 PAM PET Radioligand [11C]PF06885190 in Nonhuman Primates (NHP)

**DOI:** 10.3390/molecules28124612

**Published:** 2023-06-07

**Authors:** Sangram Nag, Ryosuke Arakawa, Zhisheng Jia, Erik Lachapelle, Lei Zhang, Kevin Maresca, Laigao Chen, Mahabuba Jahan, Timothy Mccarthy, Christer Halldin

**Affiliations:** 1 Department of Clinical Neuroscience, Center for Psychiatry Research, Karolinska Institutet and Stockholm County Council, 17164 Stockholm, Sweden; sangram.nag@ki.se (S.N.); zhisheng.jia@ki.se (Z.J.); mahabuba.jahan@foi.se (M.J.); 2 Worldwide Research, Development and Medical, Pfizer Inc., Groton, CT 06340, USA; erik.a.lachapelle@pfizer.com; 3 Worldwide Research, Development and Medical, Pfizer Inc., Cambridge, MA 02139, USA; lei.zhang3@pfizer.com (L.Z.); kevin.maresca@pfizer.com (K.M.); laigao.chen@pfizer.com (L.C.); timothy.j.mccarthy@pfizer.com (T.M.)

**Keywords:** mAChR, M4 PAM, PET, non-human primate, radiometabolites, in vivo

## Abstract

Muscarinic acetylcholine receptors (mAChR), including M4, draw attention as therapeutic targets for several neurodegenerative diseases including Alzheimer’s disease (AD). PET imaging of M4 positive allosteric modulator (PAM) allows qualification of the distribution as well as the expression of this receptor under physiological conditions and thereby helps to assess the receptor occupancy (RO) of a drug candidate. In this study, our aims were (a) to synthesize a novel M4 PAM PET radioligand [11C]PF06885190 (b) to evaluate the brain distribution of [^11^C]PF06885190 in nonhuman primates (NHP) and (c) to analyze its radiometabolites in the blood plasma of NHP. Radiolabeling of [^11^C]PF06885190 was accomplished via N-methylation of the precursor. Six PET measurements were performed using two male cynomolgus monkeys, where three PET measurements were at baseline, two after pretreatment with a selective M4 PAM compound CVL-231 and one after pretreatment with donepezil. The total volume of distribution (*V*_T_) of [^11^C]PF06885190 was examined using Logan graphical analysis with arterial input function. Radiometabolites were analyzed in monkey blood plasma using gradient HPLC system. Radiolabeling of [^11^C]PF06885190 was successfully accomplished and the radioligand was found to be stable in the formulation, with radiochemical purity exceeding 99% 1 h after the end of the synthesis. [^11^C]PF06885190 was characterized in the cynomolgus monkey brain where a moderate brain uptake was found at the baseline condition. However, it showed fast wash-out as it dropped to half of the peak at around 10 min. Change of *V*_T_ from baseline was around −10% after pretreatment with a M4 PAM, CVL-231. Radiometabolite studies showed relatively fast metabolism. Although sufficient brain uptake of [^11^C]PF06885190 was observed, these data suggest that [^11^C]PF06885190 might have too low specific binding in the NHP brain to be further applied in PET imaging.

## 1. Introduction

Muscarinic acetylcholine (MA) receptors in short mAChRs are a group of G protein-coupled receptors which can be activated by fungal toxin muscarine and play a critical role in mediating higher cognitive processing. mAChRs also control the releasing of dopamine and thereby adopt a pivotal role in human physiology [1]. In mammals, mAChRs are divided into five subtypes (M1–M5) which are expressed in both the central and peripheral nervous systems [2,3]. The subtype M4 mAChRs is mainly found in the CNS and is predominantly expressed in the striatum as well as in the hippocampus and cortex [4]. Recent studies demonstrated that the use of the M4 agonist that excites the muscarinic cholinergic system is a practical treatment for improving psychosis and behavioral disturbances in AD and schizophrenia patients [5,6,7]. Nevertheless, most of the muscarinic agonists failed in further clinical application due to adverse side effects, most probably due to the lack of sufficient receptor subtype selectivity [7,8]. There is evidence that suggests the mAChR M4 subtype is responsible for cognitive enhancement and antipsychotic-like efficacy [9]. Therefore, M4 positive allosteric modulators are believed to possess a greater possibility for target selectivity compared to an orthosteric agonist. This characteristic may lead to an improved clinical safety and tolerability profile as well as to receptor subtype selectivity [10,11,12].

Positron emission tomography (PET), a sensitive and non-invasive nuclear medicine medical imaging technique, produces powerful molecular images of the human body’s biological functions, revealing the mysteries of health and diseases. The development of PET radioligands specifically for receptors or enzymes has made it possible to evaluate the density and pharmacological action of receptors or enzymes in the brain, in addition to the receptor occupancy of therapeutic drugs [13,14,15]. Therefore, imaging of M4 PAMs using PET allows us to discover the distribution, quantification, and modulation of this receptor under physiological conditions.

**Figure 1:**
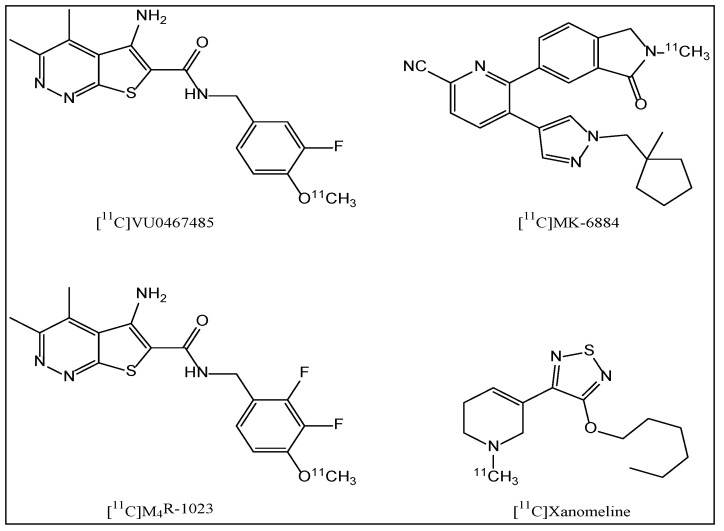
Structures of M4 PAM PET radioligands.

In recent years, several PET tracer candidates [16,17,18] (Figure 1) such as [^11^C]VU0467485, [^11^C]MK-6884, [^11^C]M_4_R-1023 and [^11^C]Xanomeline have been developed for imaging M4 PAMs using PET. Unfortunately, most of them are not suitable for use as PET ligands due to the low specific binding [17]. Cerevel Therapeutics has recently reported CVL-231, a novel, brain-penetrating M4 muscarinic positive allosteric modulator, with high affinity and selectivity [19]. M4 Muscarinic receptor occupancy of CVL-231 was further evaluated by PET in NHP [20]. Therefore, CVL-231 can serve as a suitable positive control for displacement. PF06885190 has been identified and evaluated by Pfizer as a novel M4-selective candidate with high affinity (human binding Ki = 0.16 nM, rat binding Ki = 7.15 nM) and selectivity. In this study, our aims were: (a) to radiolabel PF06885190 with C-11; (b) to evaluate the brain distribution of [^11^C]PF06885190 as a PET radioligand for M4 in nonhuman primate (NHP) and assess whether the brain uptake of [^11^C]PF06885190 can be blocked by the selective M4 PAM compound CVL-231: and (c) to analyze its radiometabolites in the blood plasma of NHP.

## 2. Results and Discussions

**Scheme 1:**
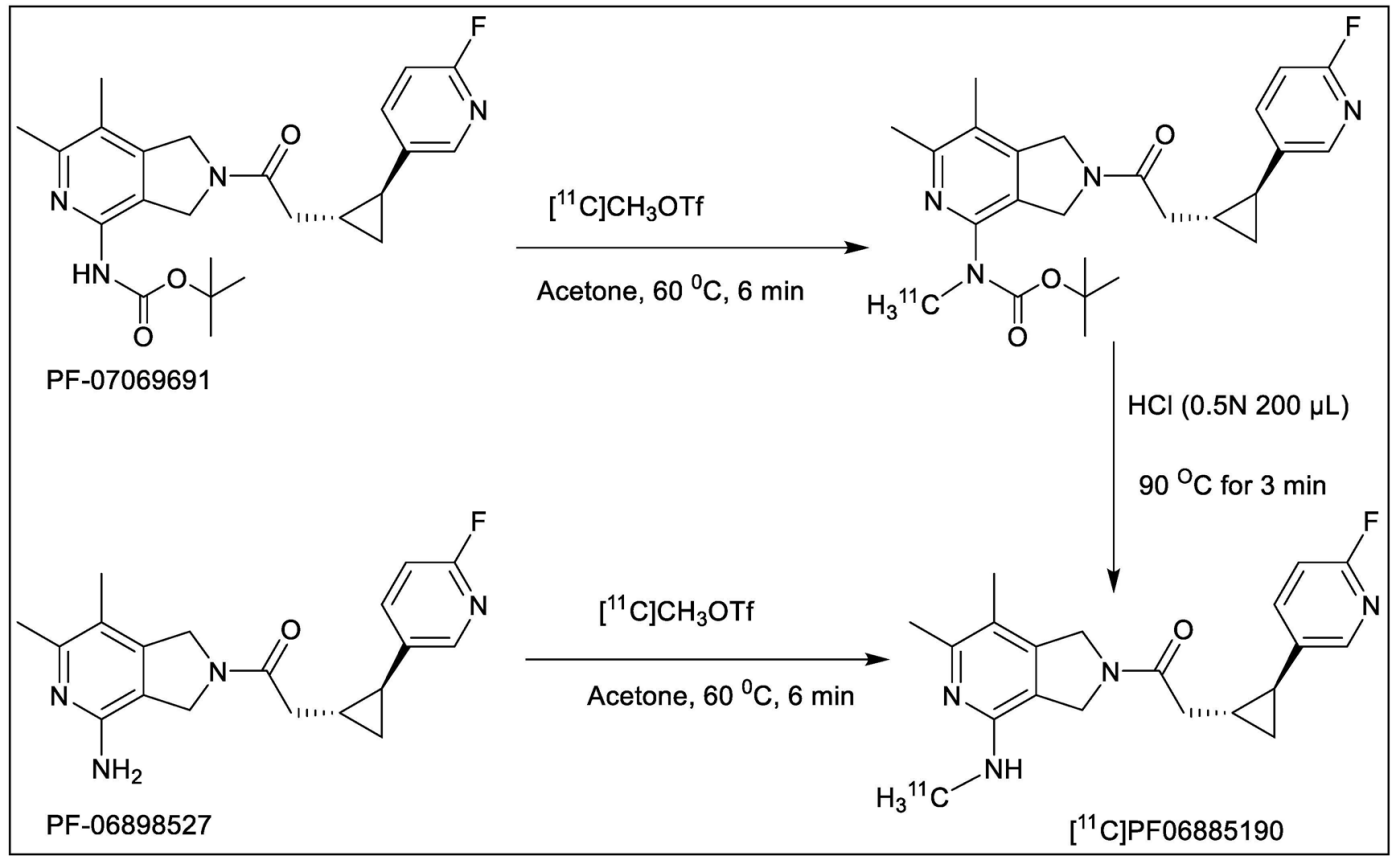
Radiosynthesis of [^11^C]PF06885190 using BOC-protected precursor (PF-07069691, **upper**) and free base precursor (PF-06898527, **bottom**).

The total time for radiosynthesis including HPLC purification, SPE isolation, and formulation of [^11^C]PF06885190 was about 35 min. The two-step N-alkylation (Scheme 1) using [^11^C]CH_3_OTf was highly reproducible and yielded >1500 MBq of the pure final product following an irradiation of the cyclotron target with a beam current of 35 μA for 15–20 min. Molar activity (MA) of the produced radioligand was in a range of 173–530 GBq/µmol at the time of the injection given to the NHP. The radiochemical purity was >99% at the end of the synthesis (EOS) and the identity of the radioligand was confirmed by co-injection of the radioligand with an authentic reference standard using a HPLC system equipped with both UV and radio detectors. Analytical HPLC method was developed and complete separation of the product PF06885190 and the precursor PF-07069691 (Figure S1A,B, Supplementary Material) was achieved. The final product, [^11^C]PF06885190, was formulated in sterile phosphate buffer solution (PBS) and was found to be stable with a radiochemical purity of more than 99% for up to 60 min.

Cyclotron target produced [^11^C]CH_4_ was utilized for the production of [^11^C]CH_3_OTf which was used as the radiolabeling agent. Fully automated production of [^11^C]PF06885190 was performed using a GE TRACERlab FX C Pro synthesizer in two steps. The first step was *N*-methylation, followed by the deprotection of the BOC-group by acid hydrolysis using hydrochloric acid. Initially, we used the free base precursor PF-06898527 for the radiolabeling. Different reaction solvents such as acetone, tetrahydrofuran, DMF, DMSO, and acetonitrile, different bases such as NaOH, KOH, and K_2_CO_3,_ various temperatures from room temperature (RT) to 100 °C, and varying amounts of precursors were explored to optimize the reaction (Table 1). Initial experiments were performed using several alkylating agents including carbon-11 labeled methyl triflate or methyl iodide ([^11^C]CH_3_OTf or [^11^C]CH_3_I) and the precursor combination (0.6 mg). While precursor reaction with [^11^C]CH_3_OTf in acetone at RT resulted in the desired product, the radiochemical yield (RCY) of <5% was low, only producing 200–250 MBq of the final product which was not enough for the in vivo evaluation.

For the next step, we decided to optimize the radiosynthesis using the BOC-protected precursor PF-07069691. Two different reaction solvents such as DMSO and acetone with various amounts of precursor, different alkylating intermediates [^11^C]CH_3_OTf or [^11^C]CH_3_I as well as different temperatures were explored to optimize the reaction. The optimal result was achieved using alkylating agents [^11^C]CH_3_OTf and precursor (0.6 mg), in acetone at 60 °C without using any base. In addition, two different synthesis modules were used to perform the synthesis, although no significant difference was observed.

**Figure 2:**
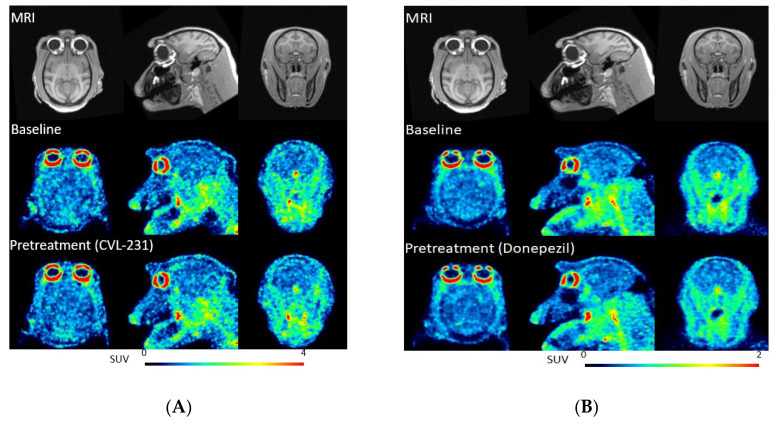
(**A**) PET images of [11C]PF06885190 co-registered with MRI in the transaxial (left), coronal (middle), and sagittal (right) projections at baseline (middle) and block with CVL-231 (bottom). Anatomical reference T1w MRI in (upper). (**B**) PET images of [^11^C]PF06885190 co-registered with MRI and averaged between 60 and 90 min in the transaxial (left), coronal (middle), and sagittal (right) projections at baseline (middle) and block with donepezil (bottom). Anatomical reference T1w MRI in (upper).

**Figure 3:**
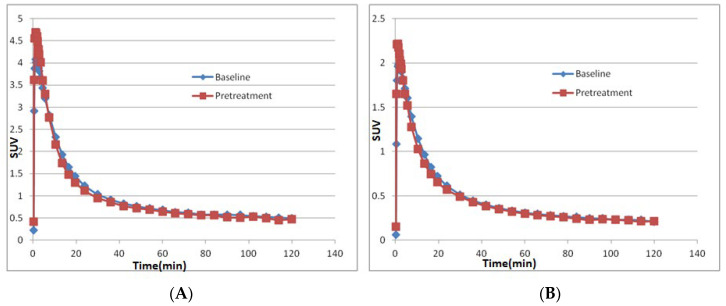
Time activity curves representing the concentration of total radioactivity in the NHP brain (**A**) at baseline and after pretreatment with CVL-231 (**B**) at baseline and after pretreatment with donepezil.

Two male cynomolgus monkeys (M1 and M2) were studied with [^11^C]PF06885190 (Table 2). At the time of injection, the injected radioactivity of [^11^C]PF06885190 was 110 ± 16 MBq and the injected mass was 0.12 ± 0.04 µg. NHP M1 underwent four PET measurements on 2 different days. PET measurements started at baseline condition after administration of [^11^C]PF06885190 followed by the second PET measurement pretreated with either CVL-231 or donepezil. Summated PET images for all two baseline and the two blocking studies, as well as T1w MRI for anatomical reference, are shown in Figure 2A,B. Whole brain uptake of [^11^C]PF06885190 was 2.0–4.1 standardized uptake value (SUV) at peak for the baseline conditions. Initially, a rapid increase in radioligand uptake was observed across the brain, with no significant difference in SUV at different brain regions. The tracer showed fast washout in all brain regions, demonstrating reversible kinetics for the tracer (Figure 3A,B). Next, CVL-231 for evaluating the blocking effect of a selective M4 PAM compound and donepezil for evaluating the increase in endogenous ACh by a cholinesterase inhibitor were tested. Time activity curves (TAC) for regional radioactivity for all four measurements involving arterial blood sampling were evaluated using kinetic 1- and 2-TC (tissue compartment) models and by Logan linear graphical analysis using 60-min data. For most measurements and brain regions analyzed, the 1-TC model was statistically preferred over the 2-TC model because the 2-TC model failed to fit in several regions. Logan graphical analysis yielded a linear phase for all measurements and regions analyzed. *V*_T_ values were obtained by the 1-TC model and the Logan method. The % decrease was calculated based on graphical analysis of *V*_T_ values obtained at baseline and after administration of CVL-231 was 12% (Table 3). No significant change was observed after the administration of donepezil. It was reported that using isoflurane anesthesia reduces the acetylcholine level in the synaptic cleft [21]. To evaluate the effect of anesthesia by isoflurane gas, two additional PET measurements were performed with a different NHP (M2), employing alternative anesthesia. In this case, anesthesia was performed by intramuscular injection of ketamine hydrochloride maintained by intravenous infusion of a mixture of ketamine hydrochloride and xylazine hydrochloride, and a PET measurement was performed with [^11^C]PF06885190 at the baseline condition, followed by pretreatment with CVL-231. The result of brain kinetics did not show any significant difference from the previous results, indicating no negative effect of isoflurane anesthesia. 

**Figure 4:**
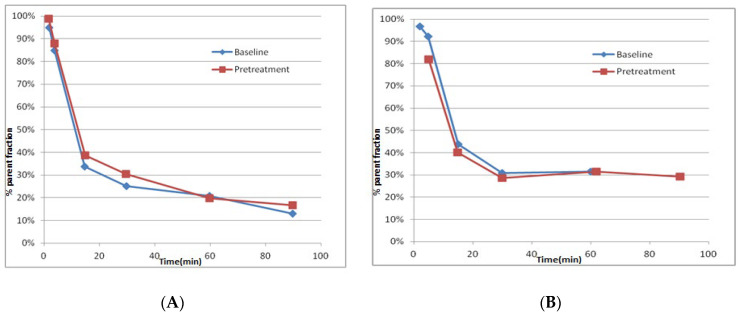
(**A**) The in vivo metabolism of [^11^C]PF06885190 is shown as the relative plasma composition at baseline condition and after pre-treatment with CVL-231) and (**B**) The in vivo metabolism of [^11^C]PF06885190 is shown as the relative plasma composition at baseline condition and after pre-treatment with donepezil.

Upon blood sampling, more than 95% of the radioactivity was recovered from plasma into acetonitrile after deproteinization. After HPLC analysis of plasma following injection, [^11^C]PF06885190 was eluted from the HPLC column at a retention time of 5.4 min. The parent compound was very abundant at 5 min, representing approximately 95% stability, and decreased to about 20% at 60 min for PET at baseline condition as well as after pretreatment with CVL-231 (Figure 4A). However, the parent compound, [^11^C]PF06885190, decreased to about 30% at 60 min for PET after pretreatment with compound donepezil (Figure 4B). Neither CVL-231 nor donepezil changed radioligand metabolism significantly. This indicated that competitive inhibition did not occur, resulting in there being no effect on brain uptake as a result of the change of input function. In addition, a few more polar radiometabolite peaks were observed under these conditions and were eluted from the HPLC column before the parent peak. The identity of [^11^C]PF06885190 was confirmed by co-injection with the non-radioactive PF06885190. 

Plasma protein binding was measured using the ultrafiltration method. The results were corrected for the membrane binding as measured with the control samples. Binding of [^11^C]PF06885190 to monkey plasma proteins at baseline and after pretreatment with donepezil were 75.2 ± 4.2% and 87.9 ± 0.3%, respectively (not available for CVL-231 due to a technical reason). Therefore, there was a measurable free fraction of [^11^C]PF06885190 in plasma: 24.8% and 12.1% at baseline and after pretreatment, respectively. The change of protein binding of [^11^C]PF06885190 was possibly caused by the competition with donepezil. Although protein binding may also affect brain kinetics, the contribution of the change of protein binding was uncertain in the present study.

## 3. Materials and Methods

### 3.1. General

Both the precursors, PF-07069691 (tert-butyl (2-(2-((1R,2S)-2-(6-fluoropyridin-3-yl)cyclopropyl)acetyl)-6,7-dimethyl-2,3-dihydro-1H-pyrrolo[3,4-c]pyridin-4-yl)carba-mate) and PF-06898527 (1-(6,7-dimethyl-4-(amino)-1,3-dihydro-2H-pyrrolo [3,4-c]pyridin-2-yl)-2-((1R,2S)-2-(6-fluoropyridin-3-yl)cyclopropyl)ethan-1-one), as well as the non-radioactive reference standard PF-06885190 (1-(6,7-dimethyl-4-(methylamino)-1,3-dihydro-2H-pyrrolo [3,4-c]pyridin-2-yl)-2-((1R,2S)-2-(6-fluoropyridin-3-yl)cyclo propyl)ethan-1-one), were synthesized by Pfizer Inc., Groton, CT, USA. All other chemicals, solvents and reagents were purchased from commercial sources. Solid-phase extraction (SPE) cartridges SepPak C18 Plus were purchased from Waters (Milford, MA, USA). The C-18 Plus cartridge was activated using EtOH (10 mL) followed by sterile water (10 mL). Liquid chromatographic analysis (LC) was performed using the software Hitachi System Manager (HSM) and the LC was equipped with a Merck Hitachi gradient pump and a MerckHitachi L-4000 variable wavelength UV-detector. The tadiosynthesis, purification and isolation of [11C]PF-06885190 was performed using two different fully automated synthesis modules, TracerMaker (Scansys Laboratorieteknik, Værløse, Denmark) and GE TRACERlab FX C Pro.

### 3.2. Synthesis of [^11^C]Methyl Triflate ([^11^C]CH_3_OTff)

[^11^C]methyltriflate ([^11^C]CH_3_OTff) was synthesized according to the previously published method [22,23,24]. The radioactive starting material, [^11^C]methane ([^11^C]CH_4_), was produced in a cyclotron target through the ^14^N(p, α)^11^C nuclear reaction of nitrogen with 10% hydrogen, with 16.4 MeV protons using a GEMS PET trace cyclotron (GE, Uppsala, Sweden). Typically, the target gas was irradiated for 30 min with a beam intensity of 35 μA. Target-produced [^11^C]CH_4_ was trapped in a cooled Porapak Q trap. [^11^C]CH_4_ was released from the Q trap and subsequently mixed with iodine vapors at 60 °C followed by a radical reaction at 720 °C in a closed circulation system. The produced [^11^C]CH_3_I was trapped in a porapak Q trap at RT and the unreacted [^11^C]CH_4_ was recirculated for 3 min. The collected [^11^C]CH_3_I was released from the Porapak Q trap by heating the trap at 180 °C with the flow of helium. [^11^C]CH_3_OTf was produced by online transfer of [^11^C]CH_3_I through a glass column packed with silver triflate at 165 °C.

### 3.3. Synthesis of [^11^C]PF-06885190

[^11^C]PF-06885190 was obtained by trapping [^11^C]CH_3_OTf at RT in a reaction vessel containing the corresponding amine precursor (PF-07069691 or PF-06898527, 1.0–1.5 mg), in acetone (400 µL) followed by heating at 50 °C for 120 s. In case of the precursor PF-07069691, the reaction mixture was additionally treated with HCl (0.5N, 200 µL) at 90 °C for 3 min to deprotect the BOC-group. After the synthesis, the residue was diluted with sterile water (2 mL) and was injected into the HPLC injection loop for purification. The HPLC loop was connected to the built-in high-performance liquid chromatography (HPLC) system equipped with a semi-preparative reverse-phase (RP) ACE column (C18, 10 × 250 mm, 5 µm particle size). The column outlet was connected with a Merck Hitachi UV detector (λ = 254 nm) (VWR, International, Stockholm, Sweden) in series with a GM-tube (Carroll-Ramsey, Berkley, CA, USA) used for radioactivity detection. A mixture of acetonitrile (15%) and trifluoroacetic acid (0.1% in water) (85%) with a flow rate of 5 mL/min was used as the HPLC isocratic mobile phase. The radioactive fraction corresponding to the desired product [^11^C]PF-06885190 was eluted with a retention time of (t_R_) 9–10 min (Figure S2, Supplementary Material) and was collected in a bottle with sterile water (50 mL). The resulting mixture was further purified by passing through a preconditioned SPE (Oasis HLB 3cc, 60 mg Sorben) cartridge. The cartridge was washed with sterile water (10 mL) and the corresponding isolated [^11^C]PF-06885190 was eluted with 1 mL of ethanol into a sterile vial containing sterile saline (9 mL) and sodium ascorbate (100 mg). The formulated product was then sterile filtered through a Millipore Millex^®^ GV filter unit (0.22 μm) for further use.

### 3.4. Quality Control (QC) and Molar Activity (MA) Determination

The radiochemical purity and stability of [^11^C]PF06885190 was determined by an analytical HPLC system coupled with an analytical XBridge column (C18 5 µm, 4.6 × 150 mm particle size), a Merck Hitachi L-7100 Pump, a L-7400 UV detector and a GM-tube for radioactivity detection (VWR International). A mixture of acetonitrile (30%) and HCO_2_NH_4_ (0.1 M aq. solution) (70%) with a flow rate of 2 mL/min was used as the HPLC isocratic mobile phase. The HPLC elution profile was monitored with an UV absorbance detector (ƛ = 254 nm) coupled to a radioactive detector (BETA-flow, Beckman, Fullerton, CA). [^11^C]PF-06885190 was eluted with a retention time of (t_R_) 4.0–4.5 min (Figure S3, Supplementary Material) and the run time of the HPLC program was 8 min. The identity of the radiolabeled compounds was confirmed by HPLC with the co-injection of the corresponding authentic reference standard. The MA was calculated by analytical HPLC following the method described previously reported [25].

### 3.5. Study Design in Non-Human Primates (NHPs), PET Experimental Procedure and Quantification

Two male cynomolgus monkeys (*Macaca fascicularis*, NHP) M1 and M2, with body weight of 7.8 kg and 7.1 kg, respectively, were studied on three different experimental days for a total of six PET measurements. Both NHPs underwent two PET measurements on the same day. The first PET measurement was performed with [^11^C]PF06885190, followed by the second PET measurement after pretreatment with either CVL-231 or donepezil. CVL-231 (0.24 mg/kg, IV) was administrated 10 min before the administration of [^11^C]PF06885190 via a constant infusion (0.0027 mg/min/kg) throughout the whole PET measurement. Donepezil (0.25 mg/kg) was administrated 30 min before the administration of [^11^C]PF06885190. Both NHPs were supplied by the Astrid Fagraeus Laboratory of the Swedish Institute for Infectious Disease Control (SMI), Solna, Sweden. The study was approved by the Animal Ethics Committee of the Swedish Animal Welfare Agency and was performed according to “Guidelines for planning, conducting and documenting experimental research” (Dnr 4820/06-600) of the KI as well as the “Guide for the Care and Use of Laboratory Animals” [26]. Anesthesia was initiated at Astrid Fagraeus Laboratory either by intramuscular injection of ketamine hydrochloride (10 mg/kg) and maintained by the administration of a mixture of isoflurane, oxygen, and medical air through endotracheal intubation, or by intravenous infusion of a mixture of ketamine hydrochloride (4 mg/kg/h) and xylazine hydrochloride (0.4 mg/kg/h). To fix the position of the head of the NHP during the course of whole PET measurement, a prototype device was used [27]. Body temperature was maintained by a Bair Hugger-Model 505 (Arizant Health Care Inc., Eden Prairie, MN 55344, USA) and monitored by an oral thermometer. ECG, heart rate, respiratory rate and oxygen saturation were continuously monitored throughout the experiments and blood pressure was monitored every 15 min.

All the PET measurements in this study were performed by using a High Resolution Research Tomograph (HRRT) (Siemens Molecular Imaging) scanner. The corresponding in-plane resolution with OP-3D-OSEM PSF was 1.5 mm full width at half-maximum (FWHM) at the center of the field of view (FOV) and 2.4 mm at 10 cm off-center directions [28]. List-mode data were acquired continuously for 123 min immediately after intravenous injection of [^11^C]PF06885190. Images were reconstructed by the ordinary Poisson-3D-ordered subset expectation maximization (OP-3D-OSEM) algorithm. Prior to each PET acquisition, a 6 min transmission scan was performed, using a single ^137^Cs source, for attenuation and scatter correction. Brain magnetic resonance imaging was performed in a 1.5-T GE Signa system (General Electric, Milwaukee, WI, USA). A T1-weighted image was obtained for co-registration with PET and delineation of anatomic brain regions. The T1 sequence was a 3D spoiled gradient-recalled (SPGR) protocol with the following settings: repetition time (TR) 21 ms, flip angle 35°; FOV 12.8; matrix 256 × 256 × 128; 128 × 1.0 mm slices; 2 NEX. The sequence was optimized for trade-off between a minimum of scanning time and a maximum of spatial resolution and contrast between gray and white matter.

For both NHPs, the radioligand [^11^C]PF06885190 was administrated with an intravenous (i.v.) injection. The regions of interests (ROIs) were delineated manually on MRI images of each NHP for the whole brain, cerebellum, caudate, putamen, thalamus, frontal cortex, temporal cortex, hippocampus, anterior cingulate cortex, posterior cingulate cortex, parietal cortex, occipital cortex, amygdala, ventral striatum and insula. The summed PET images of the whole duration were co-registered to the MRI image of the individual NHP. After applying the co-registration parameters to the dynamic PET data, the time-activity curves (TACs) of brain regions were generated for each PET measurement. Standardized uptake value (SUV) was calculated for each brain region.

Regional estimates of the total distribution volume (*V*_T_) for [^11^C]PF06885190 were obtained using one- (1-TC) and two-tissue compartment (2-TC) models, and also the Logan linear graphical method [29].

### 3.6. Radiometabolite Analysis

Radiometabolites in NHP blood plasma were analyzed following a previously published method [30]. A reverse-phase HPLC system with an UV absorbance detector (ƛ = 254 nm) coupled to a radio detector was used to determine the percentages of radioactivity corresponding to [^11^C]PF06885190 and its radioactive metabolites during whole PET measurement. Arterial blood samples (0.7–1.5 mL) were obtained using an automated blood sampling system from the monkey at different time points such as 2, 5, 15, 30 and 60 min after injection of [^11^C]PF06885190 following a previously described method [31]. Plasma was separated from collected blood by centrifuging at 4000 rpm for 2 min. The obtained plasma was diluted with 1.4 times volume of acetonitrile and then centrifuged at 6000 rpm for 4 min. The extract was separated from the pellet and was diluted with water (3 mL) before being injected into the HPLC system. The HPLC system was coupled to an Agilent binary pump (Agilent 1200 series) which was connected to a manual injection valve (7725i, Rheodyne), a 5.0 mL loop, and a radiation detector (Oyokoken, S-2493Z) housed in a shield of 50 mm thick lead. A semi-preparative reverse-phase, an ACE 5µm C18 HL column (250 × 100 mm), was used to achieve the chromatographic separation (Figure S4A,B, Supplementary Material) of the radiometabolites from the unchanged parent compound [^11^C]PF06885190 by gradient elution. Acetonitrile (A) and 0.1 M amoniumformate (B) were used as the mobile phase at flow rate 5.0 mL/min, according to the following program: 0−4.0 min, (A/B) 40:60 → 90:10 *v*/*v*; 4.0−6.0 min, (A/B) 90:10 *v*/*v*. a radioactive peak corresponding to [^11^C]PF06885190 eluted from the HPLC column was integrated and the area was expressed as a percentage of the sum of the areas of all detected radioactive compounds. To calculate the recovery of radioactivity from the system, the eluate from the HPLC column was measured in a capintec dose calibrator and divided by the amount of total radioactivity injected into the HPLC.

### 3.7. Protein Binding in Plasma

The free fraction (fp) in blood plasma was measured using an ultrafiltration method [30] with the blood sample collected before the injection of [^11^C]PF06885190. Plasma (0.4 mL) was mixed with [^11^C]PF06885190 formulation (0.04 mL, ~1 MBq) and incubated at room temperature (RT) for 10 min. To estimate the non-specific binding percent, the same process was performed on phosphate buffered saline (PBS) (0.4 mL) as a control solution. After the incubation, 0.2 mL of the plasma and PBS incubated mixtures were pipetted into ultrafiltration tubes (Centrifree YM-30, molecular weight cutoff, 30,000 Da; Millipore: Billerica, MA, USA) and centrifuged at 3800 rpm for 15 min. Equal aliquots (0.02 mL) of the ultrafiltrate (Cfree) and the plasma (Ctotal) were counted for their radioactivity with a NaI well-counter. Each determination was performed in triplicate. The free fraction was then calculated as fp = Cfree/Ctotal, and the results were corrected for the membrane binding measured with the control samples.

## 4. Conclusions

The present study demonstrated that the radioligand [^11^C]PF06885190 was efficiently labeled with carbon-11. PET imaging measurements in two cynomolgus monkeys showed high brain uptake although there was only a 10% decrease after pretreatment with specific M4 ligand CVL-231. It is suggested that [^11^C]PF06885190 might have too low specific binding to M4 PAM and might not be ideal for further application in PET measurements.

## Figures and Tables

**Table 1:** Optimization of radiosynthesis using precursors protected (PF-07069691) and free base (PF-06898527).

Precursor	Methylating Agent	Amount of Precursor (mg)	Solvent	Base	Reaction Temp (°C)	Reaction Time (min)	RCY (%)
Free-base	[^11^C]CH_3_I	0.5–1.0	Acetone	NaOH	60	5	0
Free-base	[^11^C]CH_3_I	0.5–1.0	CH_3_CN	KOH/NaOH	60	5	0
Free-base	[^11^C]CH_3_I	0.5–1.0	MeOH	KOH/NaOH	30–100	1–5	0
Free-base	[^11^C]CH_3_OTf	0.5	DMSO	NaOH	RT	3	0
Free-base	[^11^C]CH_3_OTf	0.6	DMF		RT	3	0
Free-base	[^11^C]CH_3_OTf	0.7	DMF		60	3	0
Free-base	[^11^C]CH_3_OTf	0.6	DMF		90	3	1%
Free-base	[^11^C]CH_3_OTf	0.5	DMSO		60	3	0
Free-base	[^11^C]CH_3_I	0.5	DMSO		RT	3	0
Free-base	[^11^C]CH_3_I	0.5	DMSO	KOH/NaOH	RT	5	0
Free-base	[^11^C]CH_3_OTf	0.5	DMSO		RT/60	3	0
Free-base	[^11^C]CH_3_OTf	0.7	Acetone		RT/60		0
Free-base	[^11^C]CH_3_OTf	0.6	Acetone	KOH/NaOH	RT	3	0
Free-base	[^11^C]CH_3_OTf	0.6	Acetone		60	3	2%
Free-base	[^11^C]CH_3_OTf	0.6	Acetone		RT	3	5%
Free-base	[^11^C]CH_3_OTf	1.0	Acetone		RT	3–10	5%
Protected	[^11^C]CH_3_OTf	0.6	Acetone	KOH/NaOH	RT/60	3	15%
Protected	[^11^C]CH_3_OTf	0.6–1.0	Acetone		60	3	>55%
Protected	[^11^C]CH_3_OTf	0,6	Acetone		RT	3	45%
Protected	[^11^C]CH_3_OTf	1.0	Acetone		RT	3–10	45%
Protected	[^11^C]CH_3_OTf	0.6	Acetone		75–100	3–10	25%
Protected	[^11^C]CH_3_I	0.5	DMSO	KOH/NaOH	60	5	20%

**Table 2:** Body weight of NHP, injected radioactivity, compound mass injected, and the MA at the time of injection.

NHP	SEX	Body Weight (Kg)	Administrated Radioactivity (MBq)	Molar Activity (GBq/µmol)	Administrated Mass (µg)	Condition
M1	Male	7.8	97	399	0.09	Baseline
			109	530	0.07	Pretreatment (CVL-231)
			121	258	0.12	Baseline
			131	173	0.13	Pretreatment (Donepezil)
M2	Male	7.2	112	191	0.20	Baseline
			88	275	0.11	Pretreatment (CVL-231)

**Table 3:** Calculation of V_T_ using two different models 1-TC and Logan.

	1-TC			Logan		
	Baseline	Pretreatment	%Decrease	Baseline	Pretreatment	%Decrease
**cerebellum**	2.205	1.875	15.0%	3.055	2.401	21.4%
**caudate**	2.388	2.357	1.3%	2.683	2.475	7.8%
**putamen**	2.632	2.539	3.5%	3.006	2.795	7.0%
**thalamus**	2.496	2.337	6.4%	2.778	2.367	14.8%
**frontal cortex**	2.521	2.140	15.1%	2.940	2.394	18.6%
**temporal cortex**	2.690	2.383	11.4%	2.992	2.717	9.2%
**hippocampus**	2.361	2.146	9.1%	2.595	2.341	9.8%
**anterior cing- urate cortex**	2.245	2.030	9.6%	2.503	2.293	8.4%
**posterior cing- urate cortex**	2.384	1.963	17.7%	2.723	2.225	18.3%
**parietal cortex**	2.515	2.109	16.1%	2.709	2.316	14.5%
**occipital cortex**	2.871	2.462	14.2%	3.534	2.873	18.7%
**amygdala**	2.290	2.137	6.7%	2.549	2.304	9.6%
**ventral striatum**	2.305	2.144	7.0%	2.398	2.324	3.1%
**insula**	2.617	2.314	11.6%	2.811	2.631	6.4%
**(average)**			**10.3%**			**12.0%**

## Data Availability

All the supporting data are stored at Karolinska Institutet’s archive.

## Supplementary Materials

The following supporting information can be downloaded at: https://www.mdpi.com/article/10.3390/molecules28124612/s1.

## Sample Availability

Not available.
