# Supplementary material for: Characterization of a Novel M4 PAM PET Radioligand [11C]PF06885190 in Nonhuman Primates (NHP)
Source: Molecules. 2023 Jun 7;28(12):4612. doi: 10.3390/molecules28124612 (PMC10301845; doi:10.3390/molecules28124612)
Supplement: Supplementary file 1 [file molecules-28-04612-s001.zip › molecules-2405773-supplementary.pdf]

Figure S1A

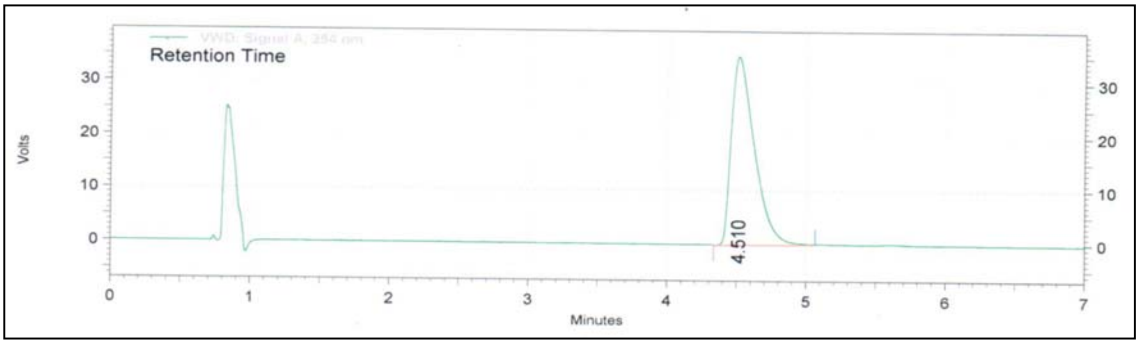

Figure S1B

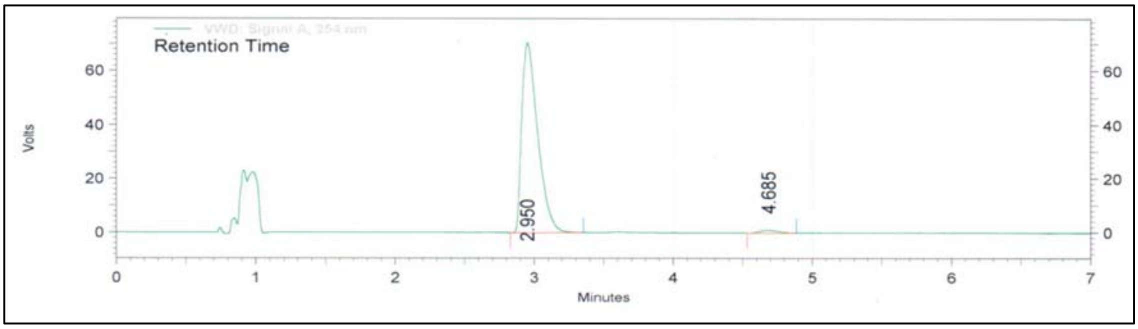

Figure S1. **A**. An HPLC chromatogram of the analysis of reference standard PF06885190 **B.** An HPLC chromatogram of the analysis of the precursor PF-07069691.

Figure S2

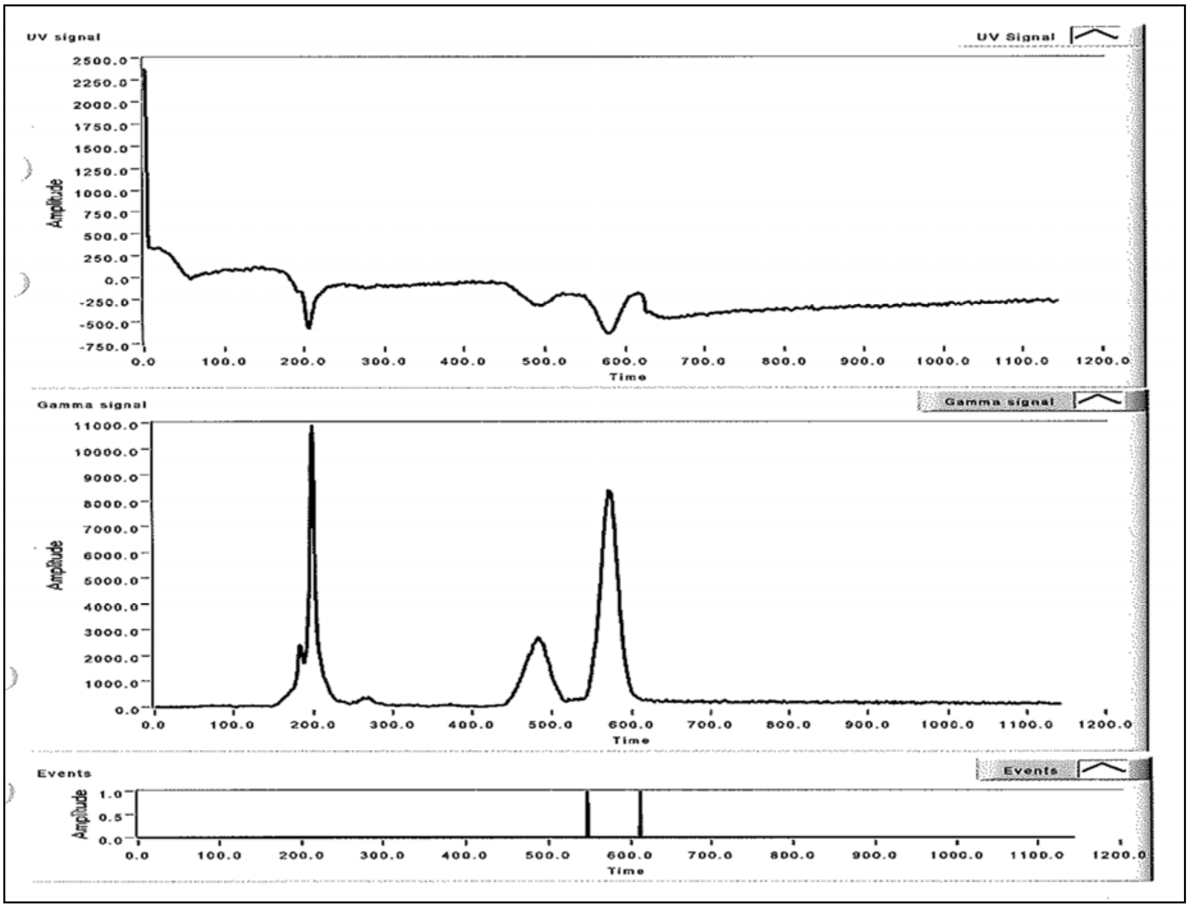

Figure S2. An HPLC chromatogram (upper: UV and bottom: radio chromatogram) of the semi-preparative purification of [^{11}C]PF-06885190

Figure S3

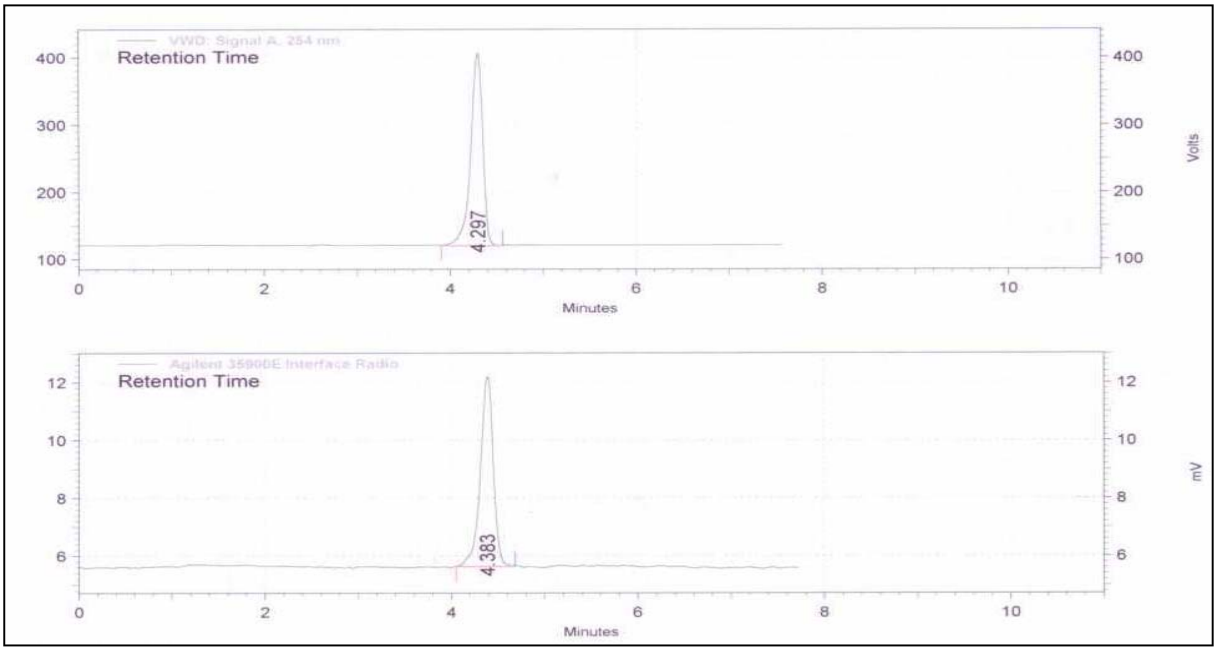

Figure S3. An HPLC chromatogram of the analysis of [11C]PF06885190 co injected with the cold reference standard PF-06885190. Upper represents the radio-chromatogram of [11C]PF-06885190 and the bottom represents the UV -chromatogram of PF-06885190.

Figure S4

A B

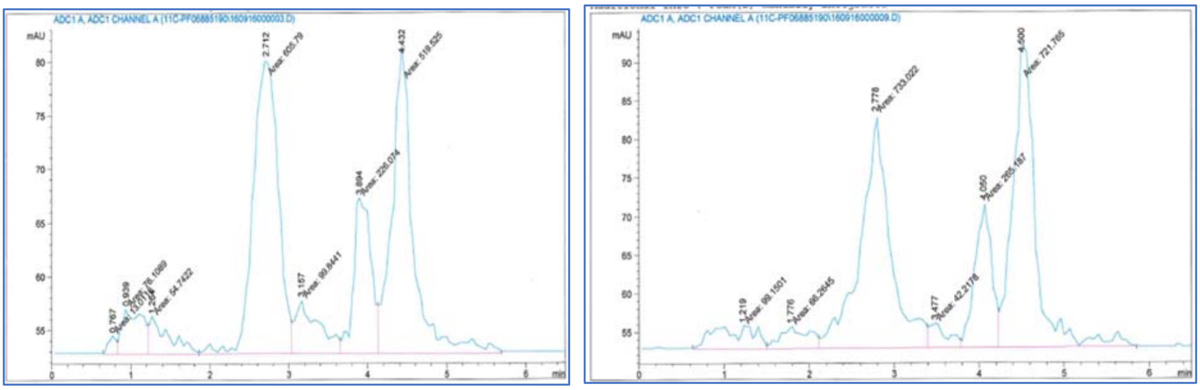

Figure S4. Radiometabolite analysis during the course of the PET measurements. It is shown as the relative plasma composition of radiometabolites and parent compound in % of total plasma radioactivity that was injected. **A.** A representative HPLC chromatogram 15 min after injection of [11C]PF-06885190 at baseline condition and **B.** A representative HPLC chromatogram 15 min after injection of [11C]PF-06885190 after pretreatment with CVL-231.
